# Oscillations and Dynamic Symbiosis in Cellular Metabolism in Cancer

**DOI:** 10.3389/fonc.2022.783908

**Published:** 2022-02-16

**Authors:** Takashi Amemiya, Tomohiko Yamaguchi

**Affiliations:** ^1^ Graduate School of Environment and Information Sciences, Yokohama National University (YNU), Yokohama, Japan; ^2^ Meiji Institute for Advanced Study of Mathematical Sciences (MIMS), Nakano, Japan

**Keywords:** metabolic oscillations, symbiosis, cancer, malignancy, heterogeneity

## Abstract

The grade of malignancy differs among cancer cell types, yet it remains the burden of genetic studies to understand the reasons behind this observation. Metabolic studies of cancer, based on the Warburg effect or aerobic glycolysis, have also not provided any clarity. Instead, the significance of oxidative phosphorylation (OXPHOS) has been found to play critical roles in aggressive cancer cells. In this perspective, metabolic symbiosis is addressed as one of the ultimate causes of the grade of cancer malignancy. Metabolic symbiosis gives rise to metabolic heterogeneities which enable cancer cells to acquire greater opportunities for proliferation and metastasis in tumor microenvironments. This study introduces a real-time new imaging technique to visualize metabolic symbiosis between cancer-associated fibroblasts (CAFs) and cancer cells based on the metabolic oscillations in these cells. The causality of cellular oscillations in cancer cells and CAFs, connected through lactate transport, is a key point for the development of this novel technique.

## Introduction

Cancers are classified into more than one hundred types owing to different organs and tissues of origin, cellular shapes, and physiological characteristics (1: https://www.cancer.net/cancer-types). The cancer type determines the grade of malignancy which is diagnosed by parameters such as five-year survival rate, prognosis, and resistance to therapy. The famous statement by Bert Vogelstein, “Cancer is, in essence, a genetic disease” (2) is widely accepted and the grade of cancer malignancy is often discussed in relation to gene expression. However, frontiers of genetic studies have not yet uncovered the causes of variable malignancies in different cancers (3).

Other studies have focused on cancer metabolism (4) and consider cancers to be metabolic diseases (5). Cancer cells are metabolically reprogrammed and enhance glycolysis even under aerobic conditions known as the Warburg effect (6, 7). The Warburg effect indicates that cancer cells produce adenosine triphosphate (ATP) and other biomolecules with high efficiency (8) which is necessary for proliferation and metastasis—one of the hallmarks of cancer (9).

Thus, the grade of cancer malignancy can be explained by the Warburg effect. If we compare cancers in different organs using the five-year survival rates, defined as the percentage of people who live longer than five years following diagnosis (10), pancreatic (8.2%) and liver (17.6%) cancers with low five-year survival rates are reported to enhance glycolysis more than breast (89.7%) and prostate (98.6%) cancers that have high five-year survival rates (11). Even in the case of cancer cells in the same type, for instance breast cancer, the cell lines with higher glycolysis rates are reported to be more malignant in terms of proliferation and metastasis (12). A review also reports that aerobic glycolysis is a crucial component of the malignant phenotype (13).

**Figure 1:**
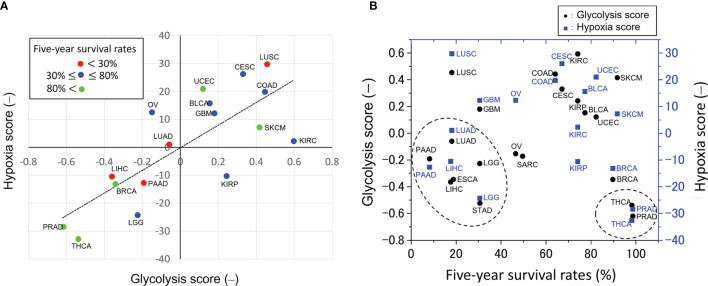
Glycolytic activities across pan-cancers. **(A)** Correlation between glycolysis and hypoxia scores obtained from gene set variation analysis (16) and mRNA-based signatures (15), respectively. This plot was made from the median values of these scores taken from the literature. The straight line is the linear regression line and the decision coefficient is *R* ^2^ = 0.601. The glycolysis score in 9,229 tumors across 25 cancer types was calculated as follows (16): first, a 22-gene expression signature (SLC2A1, HK1, HK2, HK3, GPI, PFKL, PFKM, PFKP, ALDOA, ALDOB, ALDOC, TPI1, GAPDH, PGK1, PGAM1, PGAM4, ENO1, ENO2, ENO3, PKLR, PKM and LDHA) that belongs the glycolysis core pathway was selected in each sample; second, in order to classify the glycolytic status, a gene set variation analysis (GSVA) (16) was employed to calculate the GSVA score based on the 22-gene expression signature; third, this score was scaled from -1 to 1 to yield the glycolysis score. On the other hand, the hypoxia score in 8,006 tumors across 19 cancer types was calculated as follows (15): Level 3 mRNA abundance data for all genes in a hypoxia signature developed by Buffa et al. and others (15 and references therein) were extracted from each of the cancer types. Signature-specific mRNA abundance data from all 19 cancer types were joined and scored as one cohort to compare hypoxia across cancer types. Tumors with the top 50% of mRNA abundance values for each gene in a signature were given a score +1, and tumors with the bottom 50% of mRNA abundance values for that gene were given a score -1. This procedure was repeated for every gene in the signature to generate a hypoxia score for each subject by using each signature (15). **(B)** Relation between five-year survival rates, defined as the percentage of people who live longer than five years following diagnosis (10), and the glycolysis scores as shown in **(A)**. These scores of high-grade malignant tumors of low five-year survival rates, indicated by the dotted circle, are unexpectedly very low. The scores of low-grade malignant tumors, indicated by the dotted circle, such as THCA and PRAD are low. A negative correlation between the glycolysis scores and five-year survival rates cannot be seen because the scores of the high-grade malignant tumors are too low. The abbreviations of cancer types are as follows: BLCA, bladder urothelial carcinoma; BRCA, breast invasive carcinoma; CESC, cervical squamous cell carcinoma and endocervical adenocarcinoma; COAD, colon adenocarcinoma; ESCA, esophageal carcinoma; GBM, glioblastoma multiforme; KIRC, kidney renal clear cell carcinoma; KIRP, kidney renal papillary cell carcinoma; LICH, liver hepatocellular carcinoma; LGG, lower grade glioma; LUAD, lung adenocarcinoma; LUSC, lung squamous cell carcinoma; OV, ovarian serous cystadenocarcinoma; PAAD, pancreatic adenocarcinoma; PRAD, prostate adenocarcinoma; SARC, sarcoma; SKCM, skin cutaneous melanoma; STAD, stomach adenocarcinoma; THCA, thyroid carcinoma; UCEC, uterine corpus endometrial carcinoma.

However, these studies are contradicted by big data analyses of approximately 10,000 malignant tumors using the Cancer Genome Atlas (14–16). The big data analyses obtained indexes that characterize the enhancement of the glycolytic pathway. The glycolysis score was obtained by using gene set variation analysis (GSVA) (16) and the hypoxia score was obtained by calculating mRNA-based signatures (15), see **Figure 1A** captions in detail. There is a reasonable correlation between these scores (**Figure 1A**).

In the present study, these scores were plotted as a function of the five-year cancer survival rate (10), as shown in **Figure 1B**. Notably, no negative correlation was observed between glycolysis scores and the five-year survival rate. The scores are very low for tumors with low five-year survival rates such as pancreatic (PAAD), liver (LIHC), lung (LUAD), esophagus (ESCA), glioma (LGG), and stomach (STAD) tumors. The above plot does not meet the expectation that the glycolysis scores of high-grade malignant cancers would be relatively high and that there should be a negative correlation between the glycolysis scores and the five-year survival rate. The reasons remain unknown why the pan-cancer analyses (15, 16) disagree with the widely accepted statement that “aerobic glycolysis is a crucial component of the malignant phenotype” (13).

## Significance of Mitochondrial Biogenesis and Respiration

In addition to the Warburg effect, the past two decades have witnessed a significant role of OXPHOS and a hybrid of glycolysis and OXPHOS in cancer progression and metastasis (17–21). Emerging evidence shows that mitochondrial energy pathways are reprogrammed to meet the challenges of high energy demand and biomass synthesis (20, 21). For instance, both enhanced glycolytic and increased OXPHOS activities were exhibited in highly metastatic mouse breast cancer 4T1 cells as compared with its isogenic non-metastatic 67NR cells (22). Consistently, significantly higher mitochondrial activities were found in circulating tumor cells (CTCs) derived from 4T1 cells (23) with no observable decrease in glycolytic activity. This indicates a hybrid of glycolysis and OXPHOS, which was also found in SiHa human cervix squamous cell carcinoma cells (24). Theoretically, a mathematical model based on the regulatory network of glycolysis and OXPHOS has found three stable metabolic phenotypes, the Warburg state, the OXPHOS state, and the hybrid glycolysis/OXPHOS state (19). All these studies clearly demonstrate the crucial roles of mitochondrial OXPHOS in cancer metastasis, and indicate that cancer cells are able to acquire and switch between different metabolic phenotypes.

## Metabolic Symbiosis in Cancer and Grade of Cancer Malignancy

Metabolic symbiosis is probably one the leading mechanisms, which can answer the varied malignancy in cancers (25–28). Cancer-associated fibroblasts (CAFs) are one of the candidates for establishing metabolic symbiosis with cancer cells in complex microenvironments (20, 26, 27, 29). Two types of metabolic symbiosis occur: i) cancer cells enhance their glycolytic pathway and produce lactate which is received by CAFs and oxidized in the mitochondria; ii) CAFs enhance their glycolytic pathway and produce lactate which is received by cancer cells and oxidized in the mitochondria. The first type of metabolic symbiosis has been reported in the lung (30) and colorectal (31) cancers, whereas the second type has been reported in pancreatic (32), breast (26), cervical (31) and prostate (33) cancers. In addition, the later metabolic symbiosis is significant because it suggests a modification of the Warburg effect on malignant cancers (13, 34). Metabolic symbiosis has also been proposed to occur in the brain and muscle tissue *via* lactate transport. In the brain, this is referred to as the astrocyte-neuron lactate shuttle (ANLS) (35–37).

The mechanisms of metabolic symbiosis in cancer have been proposed based on the expression levels of enzymes and transporters, such as glucose transporter 1 (GLUT1) and monocarboxylate transporter 1 (MCT1) and 4 (MCT4), in cancer cells and CAFs as determined immunohistochemical analyses (26, 31–33).

In this study, a real-time new imaging technique to visualize metabolic symbiosis between CAFs and cancer cells based on the metabolic oscillations in these cells is demonstrated. The reverse Warburg effect (38) is an essential mechanism for metabolic symbiosis in cancer. The present real-time visualization of the two-compartment tumor metabolism (26) will allow us to measure the effectiveness of anticancer therapies and facilitate more personalized cancer treatments (27).

Furthermore, the present technique has the potential to reveal the spatiotemporal dynamics of metabolic symbiosis in tumor microenvironments where populations of CAFs and cancer cells may form a metabolic network. This method can clarify the time and spatial characteristics of metabolic symbiosis between CAFs and cancer cells in tumor microenvironments. Thus, the mechanism of two-compartment tumor metabolism (26, 27) can be extended to that of multiple-compartment or network-linked tumor metabolism.

Highly malignant cancers have the plasticity to change their metabolism to glycolytic (32, 39), oxidative (27, 40), and their hybrid (20, 21) depending on the experimental conditions or microenvironments. Thus, they can acquire metabolic heterogeneities that are closely connected with proliferation, metastasis, angiogenesis, drug resistance, and other aggressive behaviors of cancer cells (40); resulting in a low five-year survival rate (**Figure 1B**).

## Metabolic Oscillations in Cancer and Other Cells

Metabolic oscillations, including glycolytic oscillations, can provide evidence of metabolic symbiosis between cancer cells and CAFs. The concentrations of all metabolites in the glycolytic pathway, such as glucose-6-phosphate, fructose 1,6-biphosphate, and pyruvate as well as ATP, adenosine diphosphate (ADP), nicotinamide adenine dinucleotide (NAD^+^), and its reduced form (NADH) oscillate in the millimolar range with periods of a few tends of seconds, which is called glycolytic oscillation. This has been primarily studied in yeasts (17, 41–44). Yeasts enhance the glycolytic pathway even under aerobic conditions by short-term phenotypic adaptation, known as the Crabtree effect (45, 46). Many types of cancer cells also exhibit Crabtree in addition to the Warburg effect which is caused by genetic mutations that enhance glycolytic activity (47).

We focused on the metabolic similarity between yeast and cancer cells (47) and succeeded in observing glycolytic oscillations in individual HeLa cervical cancer cells in monolayers and in spheroids, and DU145 prostate cancer cells in monolayers (48–51). The median frequencies were 0.0703 Hz, 0.0342 Hz, and 0.0226 Hz for HeLa cells in spheroids, in monolayers, and DU145 cells in monolayers, respectively. On the other hand, their amplitudes of NADH fluorescence were nearly the same among these cells. These oscillations directly reflect enzymatic activities in the glycolytic pathway, thus can be a useful index for evaluating the Warburg effect in cancer cells (49, 50). So far, glycolytic oscillations have not been reported in cancer patients or in healthy people, and thus it is challenging to observe their oscillations *in vivo* and to characterize them across human cancer types.

Glycolytic oscillations in cancer cells were exhibited when glucose, as the only carbon source, was added to glucose-starved cells (48, 50). On the other hand, cancers prefer alternative nutrients, such as acetate and fatty acids, in addition to glucose as the source of ATP production (52–54). However, this is observed under nutrient-rich conditions, such as in conventional tissue culture conditions *in vitro* or *in vivo*. Under the experimental conditions of glycolytic oscillations, glucose is the only source of ATP production and thus we can exclude ATP production from fatty acids, acetate, or glutamine, which are oxidized in the tricarboxylic acid (TCA) cycle.

Mitochondrial membrane potential is also known to oscillate through glucose metabolism (55–57). In pancreatic β-cells, the interaction between glycolysis and mitochondrial oxidative phosphorylation affects metabolic oscillation and plays an important role in pulsatile insulin secretion (57, 58). However, in glucose-fermenting yeasts, glycolytic and mitochondrial interactions are not fully understood. In this context, an experimental study concluded that the mitochondria had little or no regulatory effect on glycolytic oscillations (55). In contrast, other experimental and modeling studies have addressed that glycolytic and mitochondrial processes influence each other through ATP and NADH production in both glycolytic and mitochondrial pathways (59, 60).

Little is known about the glycolytic and mitochondrial interactions in cancer cells when they exhibit glycolytic oscillations (48–50). We assume that extracellular glucose is metabolized to lactate through glycolysis and fermentation without entering the TCA cycle in glucose-starved cancer cells under the experimental conditions of glycolytic oscillations (48). This is due to several different reasons: The activity of the mitochondrial pyruvate carrier (MPC) is reported to be reduced in cancer cells (61), which mainly rely on glycolysis for ATP production when glucose is the only nutrient supply; A study using a genetically encoded biosensor, which enabled monitoring of the MPC activity in living cells, showed that the level of glucose-derived pyruvate that was converted into citrate in the TCA cycle was significantly lower in cancer cells than in normal cells (61); This is further validated by another study using ^13^C NMR spectroscopy in living cancer cells (62); A review paper by McCommis and Finck (63) also reported evidence of the low activity of MPCs in various cancer cell lines and solid tumors (63); In addition, an LC-MS-based isotope tracer study showed that approximately 90–97% of pyruvate derived from extracellular glucose is metabolized to lactate whilst only 3.1–7.8% enters the TCA cycle in cancer cells (64).

## A Real-Time New Imaging Technique to Visualize Metabolic Symbiosis Between CAFs and Cancer Cells Based on Causality Between Glycolytic and Mitochondrial Oscillations

**Figure 2:**
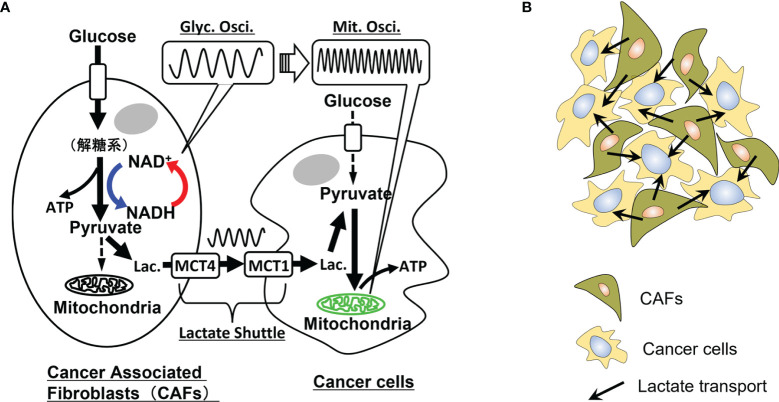
Metabolic oscillations and dynamic symbiosis between cancer cells and cancer-associated fibroblasts (CAFs). **(A)** Oscillatory symbiosis. Glycolytic CAFs enhance the glycolytic pathway and produce lactate from glucose. This lactate is secreted through monocarboxylate transporter 4 (MCT4) of CAFs, received by an oxidative cancer cell through MCT1 and metabolized in mitochondria of the cancer cells (metabolic symbiosis). Oxidative cancers, such as pancreatic and liver cancer cells, may exhibit high-glycolytic activities without the symbiosis, however, parts of the cells may exhibit the reverse Warburg effect in tumor microenvironments. We assume that causality of donor-acceptor relationships should exist between the CAFs and cancer cells metabolically connected through the lactate shuttle. Thus, if these cells exhibit metabolic oscillations, causality analysis of glycolytic oscillations in CAFs and mitochondrial membrane potential oscillations in cancer cells may directly prove the metabolic symbiosis. Glyc. Osci., glycolytic oscillations; Mit. Osci., mitochondrial membrane potential oscillations; Lac., lactate. **(B)** Lactate transport in populations of CAFs and cancer cells. In an experimental system of co-culture of CAFs and cancer cells, a cancer cell is surrounded by some CAFs and receives lactate from them. Causality analysis of their oscillatory data can determine the donor-accepter relationship between the CAFs and the cancer cell, indicating their metabolic symbiosis.

If metabolic symbiosis occurs between cancer cells and CAFs, the causality of the donor-acceptor relationship should exist through lactate transport. This metabolic causality is possibly recorded in the propagation of metabolic information from glycolytic oscillations to those in mitochondrial membrane potentials, as shown in **Figure 2A**. For example, in the case of symbiosis in pancreatic cancer, the following processes may occur chronologically: i) CAFs enhance the glycolytic pathway and may exhibit glycolytic oscillations; ii) lactate is produced from the CAFs and excreted into the extracellular space through MCT4; iii) cancer cells incorporate this lactate through MCT1 and metabolize it in mitochondria; iv) mitochondrial membrane potential may exhibit oscillations accompanied by oxidative phosphorylation.

This series of processes can be monitored by the autofluorescence from NADH and fluorescence from membrane potential sensitive dyes, such as Rhodamine 123 and tetramethylrhodamine methyl ester (TMRM) (65), respectively. A more explicit way to monitor glycolytic and mitochondrial processes independently is to use genetically encoded ATP or NADH fluorescence resonance energy transfer (FRET)-based sensors (66–68). These methods enable us to monitor cytosolic or mitochondrial ATP and/or NADH concentration levels independently.

If the above series of metabolic dynamics is observed by these imaging techniques, and also causality between glycolytic oscillations in CAFs and mitochondrial oscillations in cancer cells is proved by a statistical analysis mentioned below, then it can be the direct evidence of metabolic symbiosis between cancer cells and CAFs. This result will directly prove that highly malignant cancers, such as PAAD, LUAD, LICH, ESCA, LGG, and STAD as shown in **Figure 1B**, are able to acquire and switch between different metabolic phenotypes.

## Causality Analysis

Cancer cells and CAFs are co-cultured in an experimental system for metabolic symbiosis as shown in **Figure 2B**. In this system, it is necessary to determine the donor-acceptor relationship between cancer cells and CAFs. For example, a cancer cell can receive lactate from surrounding CAFs or metabolize glucose by itself without receiving lactate from CAFs. In such circumstances, Granger causality analysis (69), convergent cross-mapping (CCM) (70), and other statistical analyses can be used to investigate the causality between many time series of oscillatory data.

For instance, let *x*(*t*) be a time series of glycolytic oscillations in a cell of CAFs:


(1) $$x(t)=\sum_{i=1}^{P}a_{i}x(t-i)+e_{0}(t),$$

where *a_i_* is a constant, *P* is the time required to track back the data and *e*_0_(*t*) is a noise component. Equation 1 is an autoregressive model of *x*(*t*) and represents *x*(*t*) based on its past values. In addition, a time-series of mitochondrial membrane potential oscillations in a cancer cell, *y*(*t*), can be given by equations (2) and (3):


(2) $$y(t)=\sum_{i=1}^{P}b_{i}y(t-1)+e_{1}(t),$$

(3) $$y(t)=\sum_{i=1}^{P}b_{i}y(t-i)+\sum_{i=1}^{P}a_{i}x(t-i)+e_{2}(t),$$

where *b_i_* is a constant and *e*_1_(*t*) and *e*_2_(*t*) are noise components. Equation 3 expresses *y*(*t*) using its past values, as well as those of *x*(*t*). If causality exists from *x*(*t*) to *y*(*t*), the prediction accuracy of *y*(*t*) is higher in Eq. (3) than that in Eq. (2). A multi-variable vector model can be used for a system of time-series data.

## Summary

This perspective study attempted to answer the following fundamental and unresolved question: Why does the grade of malignancy differ among cancer cell types? Neither advanced cancer genome studies nor cancer-metabolic studies have completely answered this question. In addition, even the Warburg effect, one of the hallmarks of cancer, cannot answer it consistently. In the present study, the leading role of metabolic symbiosis in cancer in the tumor microenvironment was addressed. Metabolic symbiosis offers metabolic heterogeneities in cancer cells in the tumor microenvironment, resulting in resistance to anti-cancer therapies, thereby increasing the grade of malignancy. We propose that a co-culture system of cancer cells and CAFs is a good *in vitro* model. Moreover, single-cell-level metabolic oscillations and their causality analysis can directly prove metabolic symbiosis in cancer. Real-time visualization of metabolic symbiosis in cancer will allow us to measure the effectiveness of anticancer therapies and facilitate more personalized cancer treatments (27). Our symbiotic model targets metabolic interactions between CAFs and cancer cells for therapeutic strategies, including suppression of oxidative stress from cancer cells to CAFs and inhibition of metabolite transport from CAFs to cancer cells by blocking MCTs (27, 40). Breaking the network of metabolic symbiosis may result in effective anticancer therapeutic outcomes.

## Data Availability Statement

Publicly available datasets were analyzed in this study. This data can be found here: https://ourworldindata.org/cancer-death-rates-are-falling-five-year-survival-rates-are-rising.

## Author Contributions

The idea for this study was conceived by TA and developed by TY. TA wrote the manuscript in consultation with TY and both authors contributed equally to the final manuscript.

## Funding

This study was supported in part by JSPS KAKENHI Grant Numbers 19H04205 and 20K20631, a grant for a cooperative research project from YNU to TA and MEXT Promotion of Distinctive Joint Research Center Program Grant Number JPMXP0620335886 to TA and TY.

## Conflict of Interest

The authors declare that the research was conducted in the absence of any commercial or financial relationships that could be construed as a potential conflict of interest.

## Publisher’s Note

All claims expressed in this article are solely those of the authors and do not necessarily represent those of their affiliated organizations, or those of the publisher, the editors and the reviewers. Any product that may be evaluated in this article, or claim that may be made by its manufacturer, is not guaranteed or endorsed by the publisher.
